# Challenges in the PREHOSPITAL emergency management of geriatric trauma patients – a scoping review

**DOI:** 10.1186/s13049-021-00922-1

**Published:** 2021-07-23

**Authors:** Michael Eichinger, Henry Douglas Pow Robb, Cosmo Scurr, Harriet Tucker, Stefan Heschl, George Peck

**Affiliations:** 1 Major Trauma and Cutrale Perioperative and Ageing Group, Imperial College Healthcare NHS Trust, London, UK; 2 Academic Clinical Fellow in General Surgery, Imperial College Healthcare NHS Trust, London, UK; 3 Department of Anaesthesia, Imperial College Healthcare NHS Trust, London, UK; 4 Emergency Department St George’s Hospital, London, UK; 5 Department of Cardiac, Thoracic and Vascular Anaesthesiology and Intensive Care, Medical University Hospital, Graz, Austria; 6 Cutrale Peri-operative and Ageing Group, Imperial College London, London, UK

**Keywords:** Prehospital care, Triage, Geriatric trauma, Traumatic injuries, Trauma

## Abstract

**Background:**

Despite a widely acknowledged increase in older people presenting with traumatic injury in western populations there remains a lack of research into the optimal prehospital management of this vulnerable patient group. Research into this cohort faces many uniqu1e challenges, such as inconsistent definitions, variable physiology, non-linear presentation and multi-morbidity. This scoping review sought to summarise the main challenges in providing prehospital care to older trauma patients to improve the care for this vulnerable group.

**Methods and findings:**

A scoping review was performed searching Google Scholar, PubMed and Medline from 2000 until 2020 for literature in English addressing the management of older trauma patients in both the prehospital arena and Emergency Department. A thematic analysis and narrative synthesis was conducted on the included 131 studies. Age-threshold was confirmed by a descriptive analysis from all included studies. The majority of the studies assessed triage and found that recognition and undertriage presented a significant challenge, with adverse effects on mortality. We identified six key challenges in the prehospital field that were summarised in this review.

**Conclusions:**

Trauma in older people is common and challenges prehospital care providers in numerous ways that are difficult to address. Undertriage and the potential for age bias remain prevalent. In this Scoping Review, we identified and discussed six major challenges that are unique to the prehospital environment. More high-quality evidence is needed to investigate this issue further.

**Supplementary Information:**

The online version contains supplementary material available at 10.1186/s13049-021-00922-1.

## Introduction

By 2030 one in four people in the United Kingdom (UK) are expected to be older than 65 with those over 80 being the most rapidly growing demographic in our society [1]. In the last decade, national and international trauma systems have witnessed a shift in the type of trauma mechanism and age of patient admissions. Older people (≥65 years old) falling from a standing height now constitute the largest share of major trauma presentations in the UK [2]. Frailty, multi-morbidity and advanced age are widely accepted as risk factors for adverse outcomes [3]. Anatomic and physiologic changes make assessment more challenging [2, 4]. Adapting care for this vulnerable cohort is recognised as a key goal within the NHS long term plan [5]. This extends to the prehospital emergency setting where care systems differ across Europe, but the challenge of adapting to an older population remains [6]. Research specific to trauma in older people is far outweighed by that in younger cohorts, especially in the prehospital setting. This Scoping review aims to summarise the challenges in providing prehospital care to older trauma patient.

## Methods

### Search strategy

We performed a scoping literature review according to PRISMA-ScR guidelines [7]. Searches were conducted using PubMed, Google Scholar and Medline from January 2000 until April 2020 for articles written in English. The following search terms were included: prehospital, emergency, management, geriatric trauma. These terms were used rather than specific age cut-offs to ensure that all patients/papers were included, no matter what the individual paper inclusion criteria was. The terms were adjusted for the requirements of the different databases to maximise the search results (Additional File 1). Searching of references of included studies yielded additional material. Studies addressing prehospital and emergency department settings were included, regardless of the Injury Severity Score (ISS). This scoping review did not fulfil the registration criteria of PROSPERO.

### Study selection

Two independent authors (ME and HR) repeated the initial literature identification process independently from each other using identical search terms. Title and abstract screening were performed followed by full-text screening. Articles were organized using the online platform©2020 Covidence, and any conflicts were discussed and evaluated according to the inclusion criteria before consensus was reached. A third author (GP) was tasked to resolve any conflicts to limit selection bias. Articles were removed if they were updated within the set time frame, and the most recent version was included.

### Data extraction and processing

Two authors (ME and SH) performed data extraction independently. Study characteristics and relevance towards the review were evaluated and extracted. Extraction data included information and origin of the author(s), study type, study aims, age threshold number of participants. A formal quality assessment of included studies is not performed in Scoping Reviews, but the results are presented in tables with a narrative (see Additional File 1).

A narrative synthesis of included studies was performed. Statistical analysis of age cut-offs, and the distribution of included published studies over time was established using IBM® SPSS® Statistics (Version 26).

## Results

**Fig. 1:**
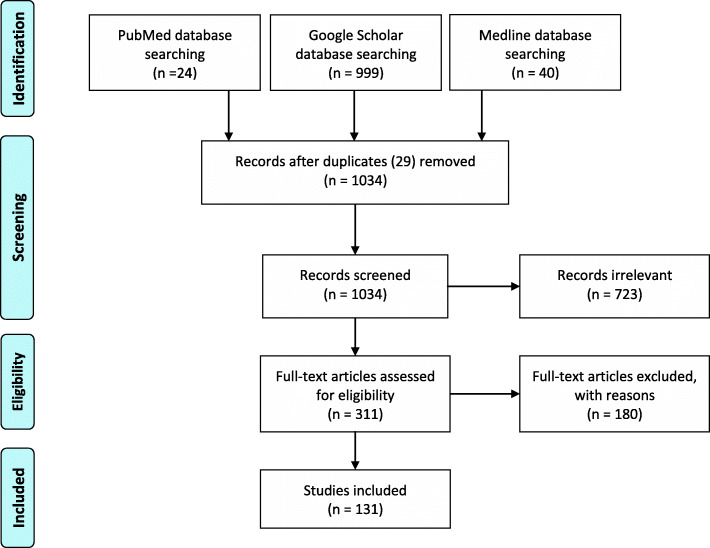
PRISMA Flow Diagram

**Fig. 2:**
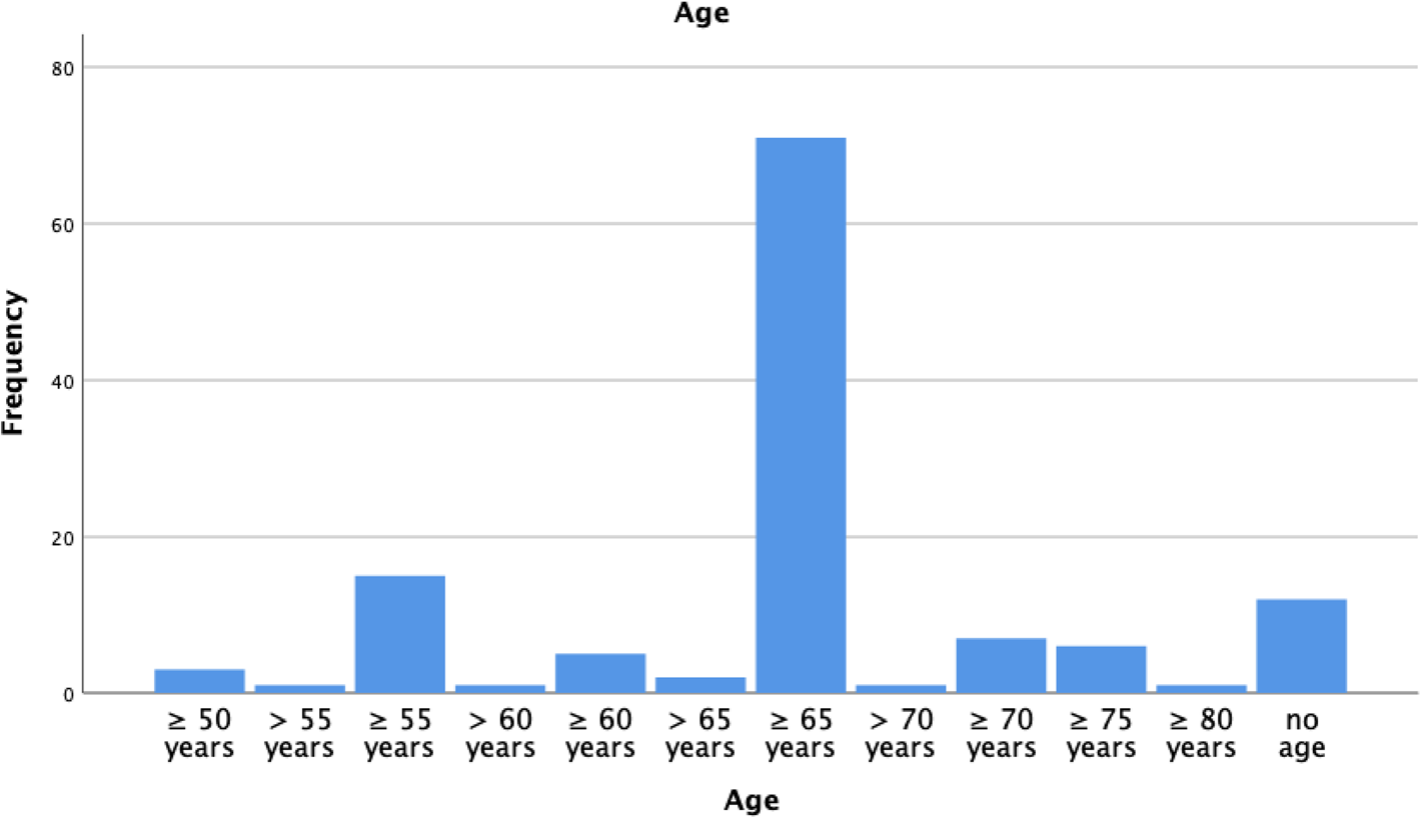
Distribution of different definitions for older age from the included studies

After 29 duplicates were excluded, 1034 publications were identified (see Fig. 1). Reasons for exclusion can be viewed in supplemental material. Additional File 1 (Table 1) gives an overview of the included studies and the study type. Additional File 1 (Table 2) shows the included studies with their characteristics, including a short narrative of the study. Many of the publications (*n* = 71/131) used a threshold of more than 65 years to define an older trauma patient, but there was no universal cut-off for older age (see Fig. 2). 93 of the 131 studies were conducted in the United States.

### Challenge 1: understanding of mechanism of injury (MOI)

#### Low-energy falls (used synonyms: ground-level falls/low-level falls/same-level falls)

An understanding of high- and low-energy mechanisms of injury and subsequent relative energy transfer is crucial for effective injury detection and trauma triage. In older adults low-energy falls are much more common, with most injuries being caused by blunt force [8]. In the presence of frailty, osteoporosis, sarcopenia and other comorbidities, these perceived low energy mechanisms can result in severe injuries [9, 10]. Falls are the most common reason (~ 60%) for severe traumatic injury and death in older adults [10–13], with over two-thirds of patients presenting more than once after a fall [14]. These events usually happen indoors [15, 16]. Musculoskeletal weakness, poor balance and slower response times to protect vital areas lead to more direct impacts to the hip or head [11]. Other frequent significant injuries include rib fractures, which are associated with increased mortality in older people [17]. Patients who have already had a fall may fear another, and therefore reduce their activities, leading to a downward spiral of rapid physiological decline [18]. A thorough evaluative falls assessment is crucial to prevent future falls and subsequent injury, especially in patients that are not transported to hospital [19], and an appreciation of the potential harm of relatively low impact falls on an older person with altered physiology and anatomy is crucial when assessing patients.

#### Road traffic collisions (RTCs)

Older people, and especially females, more commonly suffer from severe thoracic injuries after RTCs, even at low speed [11]. If involved as a pedestrian, older people are overrepresented in fatalities [11, 20, 21], and blunt aortic injuries are three times more common in this group [11]. Compared to younger patients that die on the scene, older patients are more likely to suffer from severe thoracic and pelvic injuries, in addition to traumatic brain injury and spinal cord injuries [20, 22–24]. Severe injuries are associated with the mobility status of the occupant after the crash, the angle of impact and the number of injured people [25] and ideally, these variable should be included into triage pathways [26]. Older motorcyclists sustain more injuries, especially fractures, and have more complex acute and chronic complications [27]. Interestingly, hip fractures following these incidents often cause more significant pelvic bleeding compared to younger patients, with higher massive transfusion requirements [28]. The higher prevalence of anticoagulant medications may contribute towards this [29]. An understanding of the different injury patterns in higher energy mechanisms on the older patient is key to assessment.

#### Abuse

Older people are vulnerable to physical abuse and neglect. The highest risk groups are those with cognitive impairment or communication difficulties. If injuries are inconsistent with the history provided, or other concerns are raised or suspected, then the principles and processes of Adult Safeguarding should be followed [30–32]. Patients should be supported through this process, as fear, shame, self-blame or lack of understanding of their rights mean as many as four in five cases of abuse in older people are not reported [33].

### Challenge 2: recognition of altered physiology (vital signs and monitoring)

Vital signs are used in trauma triage criteria to activate direct transfer to major trauma centres and in-hospital trauma team activation. If standard vital sign thresholds are used, this has been found to result in under-triage in older patients due to “pseudo stability”, (perceived stability when in fact the observed vital signs are not representative of the physiologic state of the patient) as the threshold for shock is lower than in younger patients [34]. A lack of recognition of haemorrhagic shock is the most frequent cause of preventable deaths in older patients with trauma [35] and so new approaches using combined values of vital signs and age, such as the age-adjusted shock index ([heart rate/systolic blood pressure] x age), have been developed with superior sensitivity and specificity [36–39]. An age-adjusted shock index of more than 50 is suspicious of occult shock [11].

#### Heart rate

Tachycardia can be masked in older people with hypovolaemic shock due to altered physiology as part of the natural aging process and the use of medications such as beta-blockers [40, 41]. Patients may be deemed “stable” in the pre-hospital setting if they present with vital signs typical for younger adults, causing under-triage [42]. There should be recognition that older patients with traumatic haemorrhage may not mount the same response as their younger counterparts, and thus suspicion of bleeding may be triggered by different heart rate parameters. Additionally, if tachycardia is present, mortality rises with heart rates greater than 90 beats per minute [43].

#### Blood pressure

Approximately a third of older trauma patients have been found to have occult hypoperfusion with a systolic blood pressure (SBP) of less than 90 mmHg, measured by lactate and base deficit [44]. Hypertension is common in older people, and thus a seemingly ‘normal’ blood pressure for a younger patient may represent significant hypotension in the older patient. An SBP of 110 mmHg has been demonstrated to be an inflection point for increased mortality in older adults [43]. Therefore, 110 mmHg may be a better threshold for identifying hypotension in trauma in older adults [11]. Using this systolic value increases overtriage only moderately, with a significant reduction in undertriage [44]. Some triage models incorporate different blood pressure parameters for Major Trauma Centre (MTC) triage in older people, but the effects on outcome with this approach are unknown.

#### Base excess and lactate

Although delays in leaving scene must be minimised, blood gas analysis before hospital arrival may contribute to patient assessment, especially in older trauma patients in whom unrecognised hypoperfusion is a common problem (20%) [44–46]. A lactate higher than 4 mmol/L or a base excess of less than − 6 mEq/L has been shown to be associated with a 40% mortality rate [47]. In those with occult or unrecognised haemorrhage, the use of lactate and base deficit in the prehospital setting may prove a useful screening tool in addition to the patient assessment.

#### Glasgow coma score (GCS) and disability

Assessment of baseline GCS is crucial for management decisions. In case of doubt [48] or if the patient suffers a drop in the GCS ≤14 post-injury, there should be a low threshold for transfer to a major trauma centre (MTC). A drop from GCS 15 to 14 has been associated with increased mortality, a higher risk for traumatic brain injury (TBI) and an increased rate of endotracheal intubation (ETI). This effect is not as significant in younger patients [32, 49, 50]. Wasserman et al. found that none of the standard scales of consciousness are reliable to detect severe TBI in older people in the pre-hospital environment [51]. The disability assessment is complex. Eye surgery or previous strokes, for example, can cause fixed pupils and can mislead pre-hospital teams [30]. Additionally, the presence of underlying cognitive impairment, dementia and delirium makes the assessment of disability particularly challenging in older adults.

Head injury is the most frequently injured body part (more than 70%) [15, 52–54] and the most under-triaged injury group in older people [55, 56]. Age-related changes in cerebral vasculature predispose to bleeding and shearing injuries. Due to the presence of cerebral atrophy, older patients with head trauma tend to have a higher initial GCS after injury, but can deteriorate quickly once the additional buffering capacity in the cranium is exceeded by haematoma expansion [13, 57]. This “talk and deteriorate” phenomenon is often seen in older patients and has increased prevalence with anticoagulant use. In addition the intracranial pressure (ICP) is often lower, and cerebral autoregulation does not function as effectively as in younger patients [58]. Older patients may not always receive the same aggressive treatment as their younger counterparts and the associated mortality in traumatic brain injury is higher [59–62].It is also important to be mindful that there is an increased incidence of chronic subdural haematomas (cSDH) with patients often presenting days after a minor trauma with a reduced GCS or other neurologic symptoms that can mimic stroke or transient ischemic attacks (TIAs) [63].

### Challenge 3: additional on-scene confounding factors

#### Delayed presentation

Older trauma patients present later to hospital. This may be for a variety of reasons. Inhabitants of nursing homes have been found to experience delays before an Emergency Medical Service (EMS) is activated after an injury. There is evidence to suggest carers may not realise or appreciate the severity of injuries prior to alerting EMS. This is also true for relatives who may adopt a ‘wait and see’ strategy to avoid the tribulations of hospitalisation [40]. These non-linear presentations complicate the triage process [64] and may contribute towards delays in accessing early appropriate care.

#### Patient assessment

Prehospital teams face a series of challenges that have an impact on patient outcomes. A quick but thorough assessment, stabilisation and rapid transport to the appropriate destination are the cornerstones of prehospital care, and may be particularly difficult to achieve in older people [48, 65]. Information from any witnesses at the scene about the mechanism of injury and description of any fall is crucial to unravel any possible medical cause for collapse leading to injury. This can be especially important for patients with a reduced GCS either due to injury or co-morbidities such as delirium or dementia. Once transferred to hospital it can be difficult to establish the physical and cognitive baseline of the patient [66] so information from the scene is critical to support handover to the receiving hospital team [30, 31].

#### Patient refusal

Older people are transported to hospital 4.5 fold more compared to younger patients with many presenting after low-level falls. However, older trauma patients are more likely to refuse conveyance to hospital after injury and one in every ten older adult does not get transported by an EMS team [67]. Compared to younger patients, there is a 25% increased likelihood of non-conveyance and this may act as a barrier to accessing care [68, 69].

Patients refuse conveyance to hospital for multiple reasons including financial worries, unwillingness to be taken to hospital for what is perceived to be a “minor” injury, or that the trauma unit or MTC is too far from home [68, 70]. The ‘scene time’ in cases of non-conveyance is 30% increased compared to patients that get transported to hospital, largely due to ensuring safety, safeguarding and thorough documentation. Non-conveyance calls are therefore resource-intensive but may not always provide visible benefit for the injured patient [67].

### Challenge 4: confounders in assessment

#### Comorbidities

##### Psychological factors

Unrecognised or severe depression has been associated with suicide attempts in older people [33]. Self-harm may be the first time older patients are diagnosed with depression. Prehospital teams will respond to these cases and should always consider team safety when hazards are involved. Such desperate situations can evolve irrationally and are an actual threat for approaching teams [71, 72].

##### Physical factors

A history of congestive heart failure, cerebral vascular accidents, hepatic diseases, renal diseases and cancer have been associated with early mortality in initially stable patients [42, 73]. Pre-existing chronic medical conditions are associated with a negative outcome. Interestingly, some conditions have been found to have a protective effect for specific injuries, for example hypertension may have a protective effect in spinal cord injury [74].

#### Medications and anticoagulation

As discussed, vital signs are influenced by medications and these play an important role in under-triage [30]. Antihypertensive drugs or psychotropic medications are associated with falls [31]. Due to the pharmacokinetic, pharmacodynamic and physiologic alterations and interactions, teams must also consider dose adjustments for treatments such as Prehospital Emergency Anaesthesia [75].

A retrospective analysis showed that beta-blockers were associated with increased mortality in injured older patients without head injury and warfarin is significantly correlated to a higher risk of intracranial haemorrhage (ICH’s) and mortality [76]. In the UK, anticoagulation prescriptions increased by 58% from 2009 to 2015 [77]. Anticoagulant and antiplatelet medications are concerning in blunt TBIs due to their predisposition towards ICH. However, the rate of delayed ICH is low, regardless of the pre-injury medication [78, 79].

In those taking anticoagulant or antiplatelet medications, minor wounds such as skin tears can lead to significant bleeding; and reversal agents are rarely available in prehospital care. Haemorrhage control is crucial as well as informing receiving teams of the agent [30, 72].By including anticoagulation within triage criteria, under-triage has been found to reduce to 2% [80], even in TBI [81]. A history of all medications, and especially anticoagulant use should be sought by all pre-hospital clinicians and, if unable to reverse in the pre-hospital setting, be handed over to the receiving team to prepare reversal agents if needed.

#### Frailty

Frailty is an independent predictor of mortality in older adults [82]. Pre-hospital frailty assessment and the training of EMS providers in the principals of frailty assessment has potential in improving triage and consequently trauma outcomes [64, 83]. Conversely, there are fears that the inappropriate use of frailty may deny access for the most frail to MTCs and therefore negatively affect outcome.

The opportunity to assess frailty in the home environment is valuable, however it can be challenging to complete extensive questionnaires. Goldstein et al. validated a care partner frailty index comprehensive geriatric assessment (CP-FI-CGA) for EMS providers although the 62 point questionnaire may have limitations in large-scale practice [84]. The trauma-specific frailty index (TSFI) is shorter with only 15 questions [85], and the Rockwood Clinical Frailty Scale is increasingly being used internationally in emergency healthcare settings [86]. Incorporating prehospital frailty screening into trauma triage criteria and patient assessment may prove valuable in the future. It is equally important that frailty screening is not used to deny access to MTCs or appropriate trauma care for patients who would otherwise have benefited from it.

#### Long lies

Worryingly, 88% of the patients are alone when they fall [87] and approximately half of all fallers are unable to get up by themselves leading to a long lie [18]. In approximately 30% of falls, patients are on the floor for over an hour. Although 15% of the fallers have a call alarm system to summon help, they are often unused (97%) [87]. Falls are the most common reason (56.9%) for rhabdomyolysis in older people and the majority of these patients (68.9%) will suffer from an acute kidney injury during their hospitalisation [88]. Pre-hospital teams should consider these potential complications before leaving this patient group at home [31], and signpost the receiving team to the potential for these complications on handover.

#### Accidental hypothermia

Both long lies and head injury can lead to hypothermia. Older people cannot maintain their temperature as well as younger people due to reduced body fat and a decreased metabolism [31, 48].. The consequences of this are coagulopathy, prolonged metabolism times of drugs and dysrhythmias [32]. Pre-hospital clinicians should attempt to minimise further heat loss where possible and anticipate and actively manage its complications [89].

#### Delirium

Delirium is associated with higher mortality and is common [33]. One frequent cause of delirium is the undertreatment of pain, explained by the concern for the potential side-effects of opioids. This hesitation may counterintuitively increase the risk of delirium [90]. Benzodiazepines should be avoided where possible in older people [33]. Pre-operative risk factors for delirium are indoor injuries, prior cognitive impairment, fever (> 37.5 °C), and prolonged waiting time to definitive treatment [91]. Many factors of trauma management, such as a stressful environment, immobilisation, or reduced perception, may increase the risk of delirium [11].

### Challenge 5: appropriate triage decision and destination

Under-triage remains the most significant challenge in the pre-hospital care of older people with trauma. This begins from the moment of injury [41, 92]. As discussed previously, EMS providers may not always recognise severe injury in older trauma patients. Altered physiology, insensitive trauma triage guidelines and the presence of cognitive impairment or delirium contribute towards this [70, 93–96]. Under-triage rates vary but may be as high as 50% [70]. Patients older than 75 with an ISS of more than 15 have been found to be 50% less likely to be triaged to a MTC compared to younger patients [97].

Whilst most literature agrees that age should be included in trauma triage criteria, there remains uncertainty on the best age cut-off to use, as evidenced in Fig. 2. Lowering the age threshold too far risks stretching trauma team resource unnecessarily and creating ‘trauma-call fatigue’, whilst raising the age threshold too high risks increasing undertriage and preventing access to appropriate and timely trauma care [55, 83, 98–101]. Many pre-hospital services do not use age-specific trauma triage criteria, and the standard adult criteria lack sensitivity to identify moderate to severe injuries in older people, principally because they focus on the mechanism of injury rather than the pre-morbid status of the individual [102–104]. Arguably, in older individuals, clinicians should focus on potential injuries rather than the solitary mechanism [105].

#### Triage destination

The Eastern Association for the Surgery of Trauma (EAST) recommends that trauma patients older than 65 years with pre-existing medical conditions should be transported to a MTC [106]. This is supported by several studies reporting reduced mortality in patients who were transported directly to a MTC [107–111]. However, several studies have shown that older trauma patients are less likely to be transferred directly to a MTC, even if they are recognised to have severe injuries [112–117]. This might be caused by an unconscious “age bias”; the discrimination of treatment solely based on age and the fear of futility of care [9, 118–121]. Some EMS providers answered in a survey that they avoid transport of older trauma patients to MTC’s as they do not want to overload it with a “simple fall” [119]. However, early geriatrician input from the time of admission has been shown to improve outcomes [122] and the length of stay (LOS) in the Emergency Department is also shorter after a trauma team activation at the MTC [123], which mitigates the adverse outcomes associated with longer emergency department length of stays in this patient group. Outcomes are also worse when the trauma team is not activated [124]. In rural areas it is has been proposed to establish trauma pathways to prevent a rise in mortality [125].

### Challenge 6: prognostication and outcome

Neither the injury severity score (ISS) nor preinjury comorbidities alone are suitable parameters to assess prognosis in older trauma patients [126]. Multiple studies have examined risk factors for early mortality in trauma patients and found that patients ≥60 years had a 4-fold increased risk of death [42, 127]. Age is associated with more complications, such as more severe organ failure [128]. Although increasing age is associated with increased mortality and morbidity, as is the male gender [129–131], management decisions should not be based on it alone. Age is not always synonymous with frailty which has also been found to play a major role in outcomes after trauma [11]. With increasing age, it becomes harder to predict the outcome after traumatic injury accurately [132]. Whilst the fatality rate is falling in younger patients (5.5%), it has not fallen (17.3%) and may even be increasing in older patients [133]. Despite this, a significant proportion of older patients who survive their initial trauma return home (67%) [134], and good long-term outcomes can be achieved, even in the oldest age groups [135]. Although a low GCS is associated with poor outcomes and is an independent risk for increased mortality [130] it is difficult to assess the outcome of TBI accurately in older people, as there are many confounders [136].

## Discussion

**Fig. 3:**
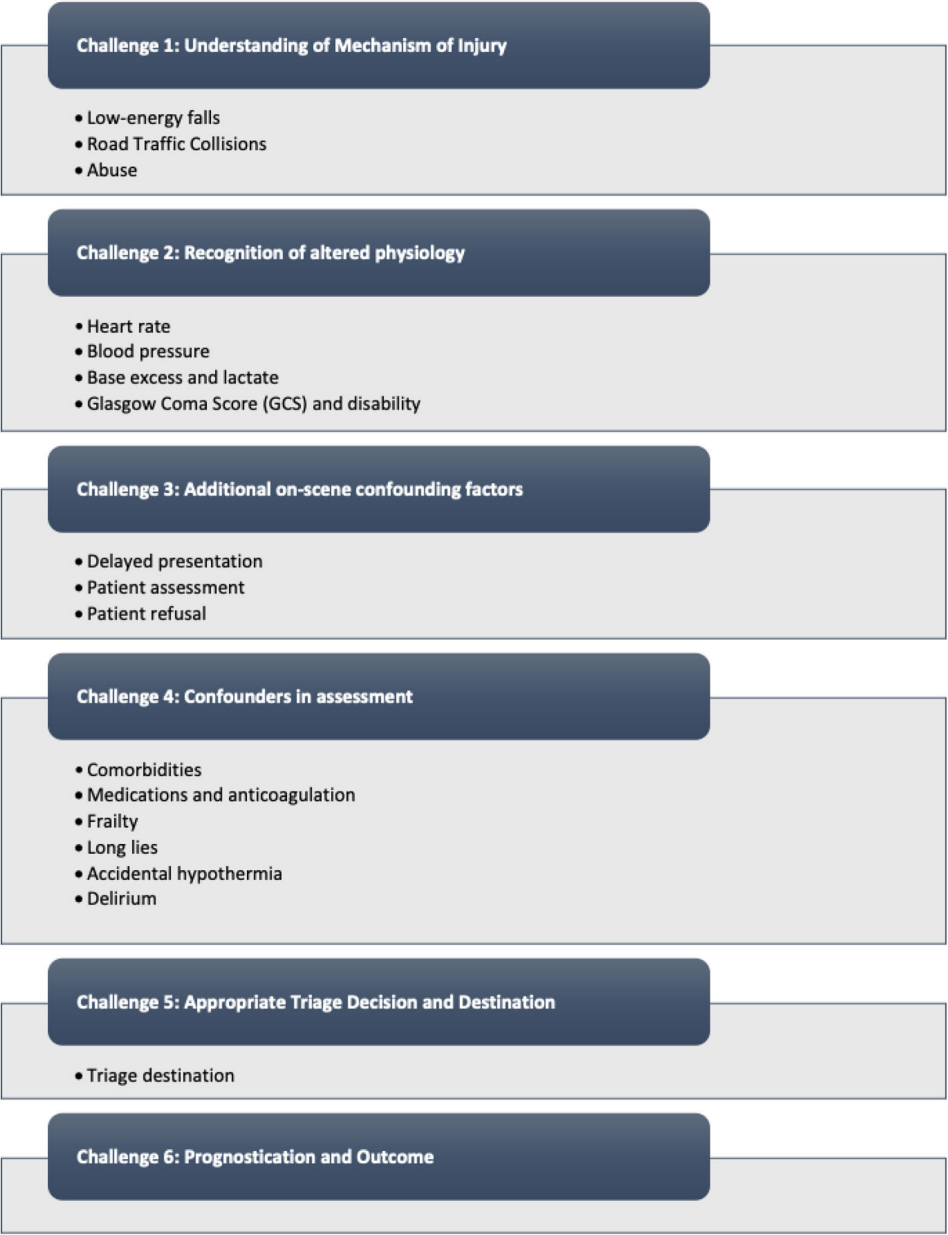
Overview of the found challenges in the management of older patients with trauma in the prehospital field

This scoping review highlights the challenges in the pre-hospital care of injured older adults. Six significant hurdles were identified that seem to be most challenging for prehospital teams (see Fig. 3). The studies included demonstrate that differences between older and younger people affect every phase of prehospital trauma care with undertriage of older adults being a crucial determinant in outcome.

The results imply that greater awareness and education of EMS providers may prove valuable in improving care for older adults. Chang et al. surveyed EMS providers establishing reasons for undertriage and found that the lack of training in the care of older patients with trauma was the most commonly stated reason, alongside unfamiliarity with older patients and age bias [119]. Another survey demonstrated that some EMS providers justified their undertriage of older patients by considering that injuries are “an expected part of the ageing process” [137]. This highlights the need for further training, education, and research in the care of this patient population, particularly in injury recognition and triage. Trauma education for EMS has focused in the past on high energy mechanisms, with low level falls in older people not receiving the same attention [9, 66]. Education of EMS providers about the principals of managing major trauma in frail older adults may need more focus to better serve the ageing population [138, 139] and lead to an increased understanding of the nuances of trauma care in older patients [140].

Support for further education is encouraged by the new geriatric trauma coalition GeriTraC [141] and other research agendas [142, 143]. A recent UK study surveyed different networks and societies to formulate research priorities for trauma of older patients and guide future research [144]. Prehospital clinicians were also involved in this survey and three of the top five research questions include prehospital care and are discussed in this review.

Key research and training questions arising from this review should focus on accurate recognition of the injured older patient, appropriate triage and current barriers to these processes. The development of geriatric standard operating procedures (SOPs), or modifications to existing SOPs based on the challenges highlighted in this review may also contribute to increased recognition, improved triage, and reduced mortality [145].

### Limitations

There is a lack of high-quality evidence. To maximise search results slightly different search terms were used according to the database requirements. MeSH terms for example include other synonyms and increase the search results but might be different to other databases.

Furthermore, most of the included studies were conducted in the United States of America. Differences in the EMS system compared to Europe limit the applicability of study findings to European healthcare systems.

## Conclusion

As far as we know, this scoping review is the first to describe the literature base and challenges in the prehospital care of older trauma patients. This review highlights six key challenges in the prehospital care of older trauma patients, but many questions remain unanswered.

The conclusions of this review can be condensed into three key aspects: 1) Prehospital management of older adults is distinct to younger patients; Altered physiology, covert mechanisms of injury, non-linear presentations and complex co-morbidities increasingly challenge prehospital teams. 2) There is a lack of high-level research in this field. 3) More education and training are needed to optimise prehospital management and improve care and outcomes.

The information gained from this review matches with the author’s own prehospital experience that managing older patients with trauma becomes increasingly complex with new difficulties that are foreign from younger counterparts.

## Supplementary Information

**Additional file 1.**

## Data Availability

All data generated or analysed during this study are included in this published article and its supplementary information files.

## Abbreviations

DICH: Delayed intracranial haemorrhage
EAST: Eastern association for the surgery of trauma
EMS: Emergency medical service
GCS: Glasgow coma scale
HR: Hear rate
ICH: Intra-cranial haemorrhage
ICP: Intracranial pressure
ISS: Injury severity score
LOS: Length of stay
MOI: Mechanism of injury
MTC: Major trauma centre
PRISMA: Preferred reporting items for systematic reviews and meta-analyses
RTC: Road traffic collision
SBP: Systolic blood pressure
SIA: Shock index – age
TBI: Traumatic brain injury
TIA: Transient ischemic attack
TSFI: Trauma-specific Frailty Index
