# Supplementary material for: Challenges in the PREHOSPITAL emergency management of geriatric trauma patients – a scoping review
Source: Scand J Trauma Resusc Emerg Med. 2021 Jul 23;29:100. doi: 10.1186/s13049-021-00922-1 (PMC8305876; doi:10.1186/s13049-021-00922-1)

## Additional material

### Detailed search strategy

PubMed
Search: prehospital management geriatric trauma (29/03/2020)

Search details: (prehospital[All Fields] AND ("organization and administration"[MeSH Terms] OR ("organization"[All Fields] AND "administration"[All Fields]) OR "organization and administration"[All Fields] OR "management"[All Fields] OR "disease management"[MeSH Terms] OR ("disease"[All Fields] AND "management"[All Fields]) OR "disease management"[All Fields]) AND geriatric[All Fields] AND ("injuries"[Subheading] OR "injuries"[All Fields] OR "trauma"[All Fields] OR "wounds and injuries"[MeSH Terms] OR ("wounds"[All Fields] AND "injuries"[All Fields]) OR "wounds and injuries"[All Fields])) AND ("2000/01/01"[PDAT] : "3000/12/31"[PDAT])

Google Scholar
Search: "prehospital" geriatric trauma management OR care -book (29/03/2020). Words must occur anywhere in the article.

Medline
Search: ("Emergency Medical Services"[Mesh] AND "Geriatrics"[Mesh]) AND "Wounds and Injuries"[Mesh] AND (("2000/01/01"[PDAT]: "3000/12/31"[PDAT]) AND English[lang]) (06/04/2020)

### Reasons for exclusion

180 studies were excluded for the following reasons:

67 Wrong setting

24 Wrong patient population

23 No full-text available

18 Outside the set time range

15 Wrong intervention

8 Book

8 Duplicate

5 Wrong indication

3 Updated article available

2 Ongoing study

2 Presentation

2 Unpublished work

2 Wrong outcomes

1 Other language than English

*Table 1: Overview of included studies and the study type*

| Study type | Included studies |
| --- | --- |
| Qualitative research | 47 |
| Cohort study | 39 |
| Text and opinion | 31 |
| Cross sectional study | 5 |
| Systematic Review | 4 |
| Review | 3 |
| Case control study | 1 |
| Consensus statement | 1 |

| **Title** | **Lead author** | **Country** | **Aim of study** | **Population description** | **Number of participants** |
| --- | --- | --- | --- | --- | --- |
| **Case control study (Level of Evidence: 3b)** | | | | | |
| Cervical Spine Fractures in Patients 65 Years and Older: A Clinical Prediction Rule for Blunt Trauma | Lawrence D. Bub | United States | To determine clinical predictors of cervical spine fracture in the elderly and to develop a clinical prediction rule to guide appropriate imaging in high-risk patients. | ≥ 65 years | 107 |
| **Cohort study (Level of Evidence: 2b)** | | | | | |
| Systolic blood pressure criteria in the National Trauma TriageProtocol for geriatric trauma: 110 is the new 90 | Joshua B. Brown | United States | To evaluate the impact of substituting an SBP of less than 110 mmHg criterion for the current SBP of less than 90 mmHg criterion within the context of the NTTP on triage performance and mortality in geriatric and adult trauma patients. | ≥ 65 years | 1555944 |
| Direct transport of geriatric trauma patients with pelvic fractures to a Level I trauma centre within an organised trauma system: impact on two-week incidence of in-hospital complications | Tabitha Garwe | United States | To evaluate the impact of the directness of transport to a Level I trauma centre on morbidity in geriatric trauma patients sustaining severe pelvic fractures | ≥ 55 years | 87 |
| Development of Statewide Geriatric Patients Trauma Triage Criteria | Howard A. Werman | United States | To describe the development of geriatric-specific field destination criteria for the state of Ohio | > 70 years | 111529 |
| Improving geriatric trauma outcomes: A small step toward a big problem | Peter M. Hammer | United States | To assess the outcomes of elderly injured patients after initiating a change in a Level I triage criteria that targeted the patient's age | ≥ 70 years | 12549 |
| Injury Severity and Comorbidities Alone Do Not Predict Futility of Care after Geriatric Trauma | David B. Duvall | United States | To objectively predict futility of care in the massively injured elderly trauma patient using easily available parameters: age, Injury Severity Score (ISS), and preinjury comorbidities. | ≥ 70 years | 570422 |
| Old age with a traumatic mechanism of injury should be a traumateam activation criterion | James M. Bardes | United States | To determine whether age≥70 years as a TTA criterion appropriately identifies patients in need of additional resources without significantly impacting overtriage rates. | ≥70 years | 739 |
| Post-trauma mortality increase at age 60: a cutoff for defining elderly? | Mackenzie Campbell-Furtick | United States | To develop an objective, data-driven definition for ‘‘elderly’’ in trauma care by evaluating mortality risk as a function of age. | ≥18 years | 872861 |
| Implementation of new standard operating procedures for geriatric trauma patients with multiple injuries: a single level I trauma centre study | Lorenz Peterer | Swizerland | To address the need for standardization of definitions and evaluating the prognostic value of injury severity scores in improving outcomes in geriatric trauma patients | ≥ 65 years | 311 |
| Falls in the elderly: a modern look at an old problem | Rondi Gelbard | United States | To evaluate the current nature of falls in the elderly | ≥ 65 years | 400 |
| Serum Lactate and Base Deficit as Predictors of Mortality in normotensive Elderly Blunt Trauma Patients | David W. Callaway | United States | To examine whether previously established markers of occult hypoperfusion, venous lactate and BD, obtained in the ED upon arrival, are associated with in-hospital mortality in normotensive elderly blunt trauma patients | ≥ 65 years | 588 |
| Triage of Elderly Trauma Patients: A Population-Based Perspective | Kristan L Staudenmayer | United States | To determine the associations between triage patterns, hospital length of stay, mortality, and costs. | ≥ 55 years | 6015 |
| Survival Benefit of Treatment at or Transfer toa Tertiary Trauma Center among Injured older adults | Tabitha Garwe | United States | To determine the difference in 30-day in-hospital mortality between moderate to seriously injured older adults treated at TCs and those treated at non-tertiary trauma centres (Non-TCs) | ≥ 55 years | 25288 |
| The age of undertriage: current trauma triage criteriaunderestimate the role of age and comorbidities in early mortality | Elizabeth R. Benjamin | United States | To identify older stable at-risk patients to potentially improve triage algorithms | ≥60 years | 1128130 |
| The value of traditional vital signs, shock index, and age-basedmarkers in predicting trauma mortality | Stevan R. Bruijns | South Africa | To establish how age, vital signs, and these markers relate to 48-hour mortality and establish a threshold for age and each of the vital signs and markers that can be used to rule in 48-hour mortality. | ≥16 | 69367 |
| Out-of-hospital and Inter-hospital Under-triage to Designated Tertiary Trauma Centers among Injured Older Adults – A 10-year Statewide Geospatial-Adjusted Analysis | Tabitha Garwe | United States | To determine the adjusted odds of treatment at a tertiary trauma center for older adult trauma patients, transported from the scene of injury and via inter-hospital transfer from a non-tertiary trauma center adjusting for the geocoded injury location, injury severity and other potentially confounding variables. | ≥ 55 years | 84930 |
| The Epidemiology of Prehospital Emergency Responses for Older Adults in a Provincial EMS System | Judah Goldstein | Canada | To quantify the rate of EMS use by older adults in a provincial EMS system, including both transport to the EDand non-transports | ≥ 65 years | 30653 |
| Age-related trends in severe injury hospitalization in Canada | Andrea D. Hill | Canada | To evaluate temporal trends in severe injury hospitalization rates, in-hospital mortality, and patterns of in-hospital bed day use following severe injury in Canada, focusing on differences between younger and older individuals | ≥ 65 years | 98937 |
| Modification of Glasgow Coma ScaleCriteria for Injured Elders | Jeffrey M. Caterino | United States | To determine if a field GCSof 14 is an appropriate cutoff to initiate transport to a trauma centre among injured elders. | ≥16 years | 92704 |
| Improving Early Identification of the High-Risk Elderly Trauma Patient By Emergency Medical Services | Craig D. Newgard | United States | To (1) define the high-risk elderly trauma patient based on prognostic differences associated with different injury patterns and (2) derive alternative field trauma triage guidelines that mesh with national field triage guidelines to improve identification of high-risk elderly patients | ≥ 65 years | 33298 |
| Evaluating Age in the Field Triage of Injured Persons | Yoko Nakamura | United States | To evaluate (1) trauma under-triage by age group; (2) the association between age and serious injury after accounting for other field triage criteria and confounders; and (3) the potential impact of a mandatory age triage criterion for field triage | ≥ 55 years | 260027 |
| Incidence of Delayed Intracranial Hemorrhage in Older Patients After Blunt Head Trauma | James A. Chenoweth | United States | What is the incidence of delayed intracranial haemorrhage in patients 55 years and older with blunt head trauma? | ≥ 55 years | 859 |
| Advanced age is associated with worsened outcomes and a unique genomic response in severely injured patients with hemorrhagic shock | Erin L Vanzant | United States | To characterize the relationship of advanced age to clinical outcomes and to transcriptomic responses after severe blunt traumatic injury with hemorrhagic shock. | ≥ 55 years | 1928 |
| A retrospective analysis of geriatric trauma patients: venous lactate is a better predictor of mortality than traditional vital signs | Kristin M Salottolo | United States | To determine whether lactate predicts mortality better than traditional vital signs in geriatric trauma patients. | ≥ 65 years | 1987 |
| Long-term survival after major trauma in geriatric trauma patients: The glass is half full | Michael D. Grossman | United States | To examine the long-term survival status of geriatric trauma patients (GTPs) after major trauma | ≥ 65 years | 145 |
| Association Between a Geriatric Trauma Resuscitation Protocol Using Venous Lactate Measurements and Early Trauma Surgeon Involvement and Mortality Risk | David Bar-Or | United States | To investigate whether implementing a geriatric resuscitation protocol that uses lactate-guided therapy with early trauma surgeon involvement is associated with lower mortality through the early recognition of occult hypoperfusion (OH) | ≥ 65 years | 1998 |
| Geriatric-Specific Triage Criteria Are More Sensitive ThanStandard Adult Criteria in Identifying Need for Trauma Center Care in Injured Older Adults | Brian Ichwan | United States | To evaluate the sensitivity of the Ohio geriatric trauma triage criteria compared with the adult triage criteria in identifying need for trauma centre care among injured older adult | ≥ 70 years | 101577 |
| Undertriage of Elderly Trauma Patients to State-Designated Trauma Centers | David C. Chang | United States | To determine if age bias plays a role in the undertriage of elderly trauma patients to TCs. | ≥ 65 years | 26565 |
| Patients Refusing Prehospital Transport Are Increasingly Likely to Be Geriatric | Peyton Holder | United States | To test the hypothesis that geriatric patients constituted an increasing proportion of persons refusing prehospital transport | ≥ 65 years | 22347 |
| Preexisting Conditions and Mortality in Older Trauma Patients | Gerald McGwin, Jr. | United States | To test the hypothesis that as the extent of physical trauma to the body increases, the role of preexisting diseases in predicting mortality will dissipate | ≥ 50 years | 129499 |
| Old Age as a Criterion for Trauma Team Activation | Demetrios Demetriades | United States | To evaluate the role of age >70 years as a criterion for Trauma Team activation | ≥ 70 years | 883 |
| Increased Trauma Activation Is Not Equally Beneficial For All Elderly Trauma Patients | Bryan W. Carr | United States | To identify the age at which an increase in the activation status of the trauma team is beneficial. | ≥70 years | 4341 |
| Trauma Team Activation for Geriatric Trauma at a Level II Trauma Center: Are the Elderly Undertriaged? | Grant S. Buchanan | United States | To evaluate whether or not geriatric trauma patients are undertriaged at a community-based level II trauma centre | ≥ 65 years | 1434 |
| Development and Validation of a Prediction Model for Prehospital Triage of Trauma Patients | Eveline A. J. van Rein | The Netherlands | To develop and validate a new prehospital trauma triage protocol to improve current triage rates. | no age | 11809 |
| The Effect of Age on Glasgow Coma Scale Score in Patients With Traumatic Brain Injury | Kristin Salottolo | United States | To investigate the effect of age on the predictive ability of the GCS for specific anatomic injury and TBI severity | ≥ 65 years | 6710 |
| Variation of Blunt Traumatic Injury with Age in Older Adults: Statewide Analysis 2011-14 | Emily Earl-Royal | United States | To assess how characteristics of blunt traumatic injuries in adults ≥ 65 vary by age. | ≥ 65 years | 38562 |
| The effect of age, severity, and mechanism of injury on risk of death from major trauma in Western Australia | Daniel M. Fatovich | Australia | To examine the association between age, mechanism of injury, and ISS on mortality for the full spectrum of major trauma | ≥ 65 years | 4411 |
| The Triage of Older Adults with PhysiologicMarkers of Serious Injury Using a State-Wide Prehospital Plan | Matthew H. Meyers | United States | To examine the impact of North Carolina's Trauma Triage Destination Plans (TTDP) implementation on the prehospital triage of geriatric patients by specific TTDP physiologic criteria | ≥ 50 years | 5345 |
| Exploring trauma recidivism in an elderly cohort | Bassan J. Allan | United States | To examine the elderly recidivist cohort at an urban trauma centre for mechanisms of repeat injury | ≥ 65 years | 6476 |
| Defining geriatric trauma: When does age make a difference? | Nicholas W. Goodmanson | United States | To identify when mortality risk increases in injured patients due to age alone, to determine whether TC care was associated with improved outcomes for these patients, and to estimate the added admissions burden to TCs using an age threshold for triage | no age | 104015 |
| **Cross sectional study (Level of Evidence: 4)** | | | | | |
| Epidemiological and Clinical Profile of Fatality in Vulnerable Road Users at a High Volume Trauma Center | Angeline Neetha Radjou | India | To study the prehospital and clinical profile of fatally injured vulnerable road users | ≥ 65 years | 193 |
| Identification of an age cutoff for increased mortality in patients with elderly trauma | Jeffrey M. Caterino | United States | To identify at what age mortality truly increases for older victims of trauma. | ≥16 years | 77197 |
| What are the differences in injury patterns of young and elderly traffic accident fatalities considering death on scene and death in hospital? | Daniela Heinrich | Germany | To answer the following questions: Do elderly RTA fatalities show a different injury pattern compared to the younger? Are injury patterns of fatalities different for death on scene and death in hospital, depending on age? Are rib cage fractures a frequent cause of death in elderly fatalities? | ≥ 65 years | 309 |
| Indoor and outdoor falls among older adult trauma patients: A comparison of patient characteristics, associated factors and outcomes | Tracy Chippendale | United States | To examine significant differences in patient characteristics, associated factors and outcomes for indoor versus outdoor falls among trauma patients | ≥ 55 years | 712 |
| Trauma in elderly people: access to the health system through pre-hospital care | Hilderjane Carla da Silva | Brazil | To identify the prevalence of trauma in elderly people and how they accessed the health system through pre-hospital care | ≥ 60 years | 2080 |
| **Consensus statement (Level of Evidence: 5)** | | | | | |
| Identification of a Neurologic Scale that Optimizes EMS Detection of Older Adult Traumatic Brain Injury Patients who Require Transport to a Trauma Center | Erin B Wasserman | United States | To identify a scale or components of a scale that optimize detection of older adult TBI patients who require transport to a trauma centre, regardless of mechanism. | ≥ 55 years | - |
| **Review (Level of Evidence: 5)** | | | | | |
| Older people and major trauma | Elaine Cole | UK | To give a comprehensive overview of research literature pertaining to older people and major trauma, with a view to making recommendations for policy, practice, research and education | ≥ 75 years | - |
| Prehospital factors associated with severe injury in older adults | Linda J. Scheetz | United States | To review prehospital factors associated with severe injuries amongst older adults. | ≥ 50 years | - |
| Geriatric Trauma | A Mitra | India | To evaluate alterations with ageing in the trauma population | ≥ 65 years | - |
| **Qualitative research (Level of Evidence: 4)** | | | | | |
| Injury in the aged: Geriatric trauma care at the crossroads | Rosemary A. Kozar | United States | To survey the membership of the AAST to better understand the current conditions under which hospitalized GTPs are receiving care and to enumerate the major problems associated with the care of GTPs and to suggest potential solutions to the identified problems. | ≥ 65 years | - |
| Shock index predicts mortality in geriatric trauma patients: An analysis of the National Trauma Data Bank | Viraj Pandit | United States | To identify the utility of shock index in predicting outcomes in geriatric trauma patients | ≥ 65 years | 485595 |
| The impact of advanced age on trauma triage decisions and outcomes: A statewide analysis | Ryan Lehmann | United States | To evaluate the triage patterns of elderly versus nonelderly patients in the state of Washington, as well as to analyze the impact of these triage practices on the morbidity and mortality of these patients | > 65 years | 51227 |
| Preinjury Beta Blockers are Associated With Increased Mortality in Geriatric Trauma Patients | Todd Neideen | United States | To test the hypothesis that pre-trauma beta-blocker use would increase mortality | ≥ 65 years | 1479 |
| Effectiveness of Prehospital Trauma TriageGuidelines for the Identification of Major Trauma in elderly Motor Vehicle Crash Victims | Linda J. Scheetz | United States | To examine the sensitivity and specificity of one state’s prehospital trauma triage guidelines for adults, with a particular focus on the triage of elderly persons | ≥ 65 years | 2063 |
| Opportunities for improved trauma care of the elderly - A single center analysis of 2090 severely injured patients | Amadéa Schönenberger | Switzerland | To identify shortcomings and opportunities for improvement in the treatment of elderly trauma patients | ≥ 75 years | 2090 |
| The Value of the Trauma Mechanism in the Triage of Severely Injured Elderly | Johanna M. M. Nijboer | The Netherlands | To evaluate whether current trauma triage criteria are appropriate in severely injured elderly patients. | ≥ 55 years | 191 |
| Do Trauma Patients Aged 55 and Older Benefit from Air Medical Transport? | Howard A. Werman | United States | To evaluate mortality benefits of air transport in adult trauma patients55years of age | ≥ 55 years | 8421 |
| Using Crash Scene Variables to Predict the Need for Trauma Center Care in Older Persons | Linda J. Scheetz | United States | To identify crash scene information readily available to EMS personnel that could be used to predict severe and moderate injury in older persons | ≥ 65 years | 7883 |
| Evaluation of major trauma in elderly patients – a single trauma center analysis | Samo Kocuvan | Slovakia | To examine the group of elderly polytrauma patients treated at a Slovenian trauma centre, specifically with respect to mechanisms of injury, injury severity and distribution of injuries; to identify morbidity and mortality rates and compare these to the younger population, and to determine the factors that have the most considerable impact on the treatment results | ≥ 65 years | 532 |
| Factors Associated With Trauma Center Use for Elderly Patients With Trauma | Renee Y. Hsia | United States | To estimate the likelihood of trauma centre admission for injured elderly patients with trauma, determine trends in trauma centre admissions, and identify factors associated with trauma centre use for elderly patients with trauma | ≥ 65 years | 430081 |
| Riding into the golden years: injury patterns and outcomes of advanced-age motorcycle trauma | Sydne Muratore | United States | To characterize injury, complications, and outcomes for older riders after motorcycle accidents due to the rising trend in advanced-age motorcyclists | ≥ 55 years | 432 |
| Survival Advantage for Elderly Trauma Patients Treated in a Designated Trauma Center | Etienne E. Pracht | United States | To analyze the effectiveness of designated trauma centers (DTCs) in Florida concerning reduction in the mortality risk of severely injured elderly trauma victims. | ≥ 65 years | 57976 |
| Differences in Survival, Length of Stay, and DischargeDisposition of Older Trauma Patients Admitted toTrauma Centers and Nontrauma Center Hospitals | Linda J. Scheetz | United States | To examine the relationship of level of care (trauma centre, non-trauma centre hospitals) on three outcomes: survival, length of stay, and discharge disposition. | ≥ 65 years | 1418 |
| Preventable and Potentially Preventable Deaths in Severely Injured Elderly Patients: A Single-Center Retrospective Data Analysis of a German Trauma Center | Carsten Schoeneberg | Germany | To focus on preventable or potentially preventable deaths, and on patterns of errors in severely injured elderly patients, a group that is under-represented in the literature. | ≥ 75 years | 108 |
| Geriatric hospitalizations in fall-related injuries | Cheng-Shyuan Rau | Taiwan | To investigate the injury pattern, severity, and mortality of elderly patients hospitalized for treatment of trauma following fall accidents | ≥ 65 years | 16548 |
| Anticoagulated trauma patients: a level i trauma center’s response to a growing geriatric population | Mark D. Mason | United States | To examine if undertriage rates can be lowered when a limited trauma team response for anticoagulated older adults is implemented in the ED at a Level I trauma center | ≥ 55 years | 318 |
| Clinical Outcome and Management for GeriatricTraumatic Injury: Analysis of 2688 Cases in the Emergency Department of a Teaching Hospital in Taiwan | Meng-Yu Wu | Taiwan | To investigate the pre-hospital and in-hospital data to analyze clinical outcomes and the prediction scores for the elderly and younger patient groups | ≥ 65 years | 2688 |
| Injury patterns and outcomes associated with elderly trauma victims in Kingston, Ontario | Rob Gowing | Canada | To characterize the common injuries incurred by elderly trauma victims and to identify the most frequent complications and outcomes | ≥ 65 years | 125 |
| It is time for a change in the management of elderly severely injured patients! An analysis of 126,015 patients from the TraumaRegister DGU | Christopher Spering | Germany | To look for differences in the management of severely injured elderly patients compared to younger age groups and to evaluate their potential impact on the outcome. | ≥ 60 years | 126015 |
| Role of trauma team activation in poor outcomes of elderly patients | Alexander E. St. John | United States | To investigate the role of trauma team activation in outcomes of elderly trauma patients | ≥ 65 years | 10033 |
| Comparative analysis of the characteristics of traumas suffered by elderly and younger patients | José Gustav Parreira | Brazil | To identify the characteristics of traumas suffered by elderly people by comparing their variables with a group of trauma patients who were not elderly | ≥ 60 years | 2075 |
| Comparison of type and severity of major injuries among undertriaged and correctly triaged older patients | Linda J. Scheetz | United States | To describe the injury types, injury severity, and short-term survival outcomes of undertriaged older adults injured in motor vehicle collisions, compared to a correctly triaged cohort, to identify any unique injuries that might be associated with undertriage. | ≥ 65 years | 66445 |
| Prehospital Triage of Older Adults with Head Injury: A Retrospective Study of the Impact of Adding “Anticoagulation or Antiplatelet Medication Use” as a Criterion | Daniel K. Nishijima | United States | To describe the characteristics and outcomes of older adults with head trauma | ≥ 55 years | 2110 |
| Early neurological deterioration in older adults with traumatic brain injury | Linda J. Scheetz | United States | To determine the proportion of older adults with TBIs whose GCS score deteriorated from prehospital to ED assessment, factors associated with a decrease in GCS score, and the association of the magnitude of GCS score deterioration with the anatomic severity of the brain injury | ≥ 65 years | 144678 |
| Triaging older major trauma patients in the emergency department: an observational study | William Lukin | Australia | To examine whether older major trauma patients were less likely to be assigned an emergency triage category on arrival to the emergency department after controlling for relevant demographics, injury characteristics and injury severity | no age | 6923 |
| Disparities in Trauma Center Access of Older Injured Motor Vehicular Crash Occupants | Gabriel E. Ryb | United States | To evaluate whether older injured motor vehicular crash occupants’ access to trauma centres reflects the lower threshold included in the triage recommendations or whether a disparity exists where those older than a certain age would be less likely to be transported to a trauma centre. | > 60 years | 7894940 |
| Road traffic injuries in the elderly | W Y Yee | Singapore | To investigate the injury pattern of elderly victims involved in motor vehicle collisions | ≥ 65 years | 1539 |
| Injury patterns, severity and outcomes among older adults who sustained brain injury following a same-level fall: A retrospective analysis | Linda J. Scheetz | United States | To identify the incidence and types of brain injuries; classify brain injury severity; identify additional injuries; and identify predictors of length of stay, mortality and trauma centre admission | ≥ 65 years | 3331 |
| Delta Alerts: Changing Outcomes in Geriatric Trauma | Lynn L. Wiles | United States | To evaluate a decrease in M&M by early identification of injuries, proper speciality group referrals, and coordination of interdisciplinary care | ≥ 65 years | - |
| Trends in the accuracy of older person trauma triage from 2004 to 2008 | Linda J. Scheetz | United States | To examine triage accuracy before and after the 2006 revisions of the American College of Surgeons Committee on Trauma (ACS-COT) Field Triage Decision Scheme | ≥ 55 years | 954720 |
| Validation of the Shock Index, Modified Shock Index, and Age Shock Index for Predicting Mortality of Geriatric Trauma Patients in Emergency Departments | Soon Yong Kim | South Korea | To validate the power of the SI, MSI, and Age SI in the prediction of mortality in geriatric trauma patients | ≥ 65 years | 45880 |
| Severely Injured Geriatric Population: Morbidity, Mortality, and Risk Factors | Noura Labib | Canada | To determine whether pre-existing comorbidities had an effect on outcome of severely injured older trauma patients | ≥ 65 years | 283 |
| Normal Presenting Vital Signs Are Unreliable in Geriatric BluntTrauma Victims | Daithi S. Heffernan | United States | To examine the effect of derangements in vital signs on presentation in a geriatric population on survival | ≥ 65 years | 4275 |
| Trauma Center versus Non-Trauma Center admissions in adult Trauma victims by age and gender | Linda J. Scheetz | United States | To compare the admission types and appropriateness of admission of adults with differing levels of injury severity, based on patient age and gender. | ≥ 65 years | 5712 |
| Relationship of Age, Injury Severity, Injury Type, ComorbidConditions, Level of Care, and survival among Older Motor Vehicle Trauma Patients | Linda J. Scheetz | United States | To compare age, injury severity, injury types, selected comorbidities, level of care (at trauma centre and non–trauma centre hospitals), and survival among older motor vehicle trauma patients | ≥ 65 years | 1478 |
| Geriatric Trauma Practice Management Guideline (Update) Eastern Association for the Surgery of Trauma | James Forrest Calland | United States | To create a guideline for the management of geriatric trauma patients | > 65 years | - |
| Trauma Triage: The Effect of Age on EMS Provider Clinical Judgment and Prehospital Guidelines for Transportation of Injured Patients | Courtney Marie Cora Jones | United States | To assess different potential reasons for the undertriage of older adults to trauma centres | ≥ 70 years | - |
| The geriatric trauma patient: A neglected individual in a mature trauma system | Michael A. Horst | United States | To determine the rate of geriatric undertriage to TCs within a mature trauma system and hypothesized that there would be variation and clustering of the geriatric undertriage rate within a mature trauma system because of the admission of geriatric trauma patient to non-trauma centres | ≥ 65 years | 169962 |
| Delayed intracranial hemorrhage in elderly anticoagulated patients sustaining a minor fall | Nolan Mann | United States | To evaluate the incidence of delayed intracranial haemorrhage detected by head computer tomography among an elderly population on pre-injury anticoagulant or antiplatelet therapy | ≥ 65 years | 218 |
| Octogenarians and motor vehicle collisions: Postdischarge mortality is lower than expected | Kathryn S. Soba | United States | To evaluate possible associations between MVC injury severity, injury patterns, and hospital course with long-term mortality outcomes in octogenarian patients who survived their initial hospitalization at a Level I trauma centre. | ≥ 80 years | 199 |
| Efficacy of Prehospital Criteria in IdentifyingTrauma Patients Susceptible to Undertriage | Sawyer G. Smith | United States | To determine if other clinically relevant prehospital criteria could be used to accurately identify patients who are more likely to be undertriaged. | no age | 3123 |
| Accuracy of algorithms to predict injury severity in older adults for trauma triage | Thomas Hartka | United States | To quantify the change in accuracy of a regression-based automatic injury prediction model for older adults and examine the effect of this change on rates of appropriate triage | ≥ 65 years | 24577 |
| Identifying Life-Threatening Shock in the Older Injured Patient: An Analysis of the National Trauma Data Bank | Ben L. Zarzaur | United States | To determine the ability of SI and age SI to predict early mortality after blunt injury | > 55 years | 507262 |
| Trauma and Falls in the Elderly | Miriam T. Aschkenasy | United States | To give an overview of trauma in the Elderly | ≥ 65 years | - |
| Older Adult Falls in Emergency Medicine - 2019 Update | Christopher R. Carpenter | United States | To give an overview of hazards in the emergency management of older adults after falls | ≥ 65 years | - |
| Geriatric Outcomes Are Improved by a Geriatric TraumaConsultation Service | William F. Fallon | United States | To assess the effect of implementing a geriatric trauma consultation on the outcome of older trauma patients. | ≥ 65 years | 285 |
| **Systematic Review (Level of Evidence: 3a)** | | | | | |
| Accuracy of prehospital triage protocols in selecting severely injured patients: A systematic review | Eveline A.J. van Rein | The Netherlands | To evaluate the current literature on all available prehospital trauma triage protocols and determine accuracy of protocol-based triage quality in terms of sensitivity and specificity | no age | - |
| Global burden of road traffic accidents in older adults: A systematic reviewand meta-regression analysis | Boon Hong Ang | Malaysia | To estimate the burden of road traffic accidents and death among older adults | ≥ 65 years | - |
| Is age associated with emergency medical service transport to a trauma centre in patients with major trauma? A systematic review | Elizabeth Brown | Australia | To answer the question: are older patients with major trauma more or less likely to be transported to a trauma centre by emergency medical services (EMS) than younger patients? | no age | - |
| Missed opportunities: Under-detection of trauma in elderly adults involved in motor vehicle crashes | Jenelle M. Weber | United States | To answer the question if older adults involved in MVCs are appropriately assessed and treated. | ≥ 65 years | - |
| **Text and opinion (Level of Evidence: 5)** | | | | | |
| Improving adjustments for older age in prehospital assessment and care | Marius Rehn | Norway | To comment on necessary adjustments for older age in prehospital care | no age | - |
| Evolving Prehospital, EmergencyDepartment, and “Inpatient” Management Models for Geriatric Emergencies | Christopher R. Carpenter | United States | To give an overview of inpatient management models for older adults | ≥ 65 years | - |
| Geriatric trauma | Sasha D. Adams | United States | This review explores recent literature on geriatric trauma, including triage criteria, assessment of frailty, fall-related injury, treatment of head injury complicated by coagulopathy, goals of care, and the need for ongoing education of all surgeons in the care of the elderly. | ≥ 65 years | - |
| Geriatric Trauma Care: Challenges Facing Emergency Medical Services | Isaac Chu | United States | To highlight problems of EMS with geriatric trauma care | - | - |
| Geriatric Trauma: Special Needs for a Special Population | Barbara Pudelek | United States | To describe differences in the management of older trauma patients | ≥ 65 years | - |
| High Yield Research Opportunities in Geriatric Emergency Medicine: Prehospital Care, Delirium, Adverse Drug Events, and Falls | Christopher R. Carpenter | United States | To map potential areas of research in Geriatric Emergency Medicine | no age | - |
| ‘Major trauma’: now two separate diseases? | Timothy J Coats | UK | To define two different types of major trauma patients (high energy trauma and low energy trauma) with different needs. | - | - |
| Evidence-Based Care of Geriatric Trauma Patients | Steven E. Brooks | United States | To review the different physiology of geriatric trauma patients, the treatment of specific injuries and other considerations in the management. | ≥ 65 years | - |
| High-Energy Skeletal Trauma in the Elderly | Julie A. Switze | United States | To review the different mechanism of injury, treatment within the trauma system, the outcomes and the operative treatment of the elderly trauma patient. | ≥ 60 years | - |
| Integrated Health Care Management of Moderate to Severe TBI in Older Patients—A Narrative Review | Rahel Schumacher | Switzerland | To summarise and critically evaluate important aspects of the health care management of older trauma patients in covering the entire pathway from prehospital care to rehabilitation and beyond. | ≥ 65 years | - |
| Traumatic Brain Injury in Older Adults: Epidemiology, Outcomes, and Future Implications | Hilaire J. Thompson | United States | To address TBI in older adults and review different management strategies, mechanisms of injury and underlying health conditions. | ≥ 65 years | - |
| The Tragically Hip - Trauma in Elderly Patients | Katrin Hruska | Sweden | To explain problems with geriatric trauma patients | no age | - |
| The Changing Landscape of Trauma Care, Part 1 | Ashley Menne | United States | To present challenges in treating the increasing numbers of old trauma patients | ≥ 65 years | - |
| The Role of Emergency Medical Services in Geriatrics: Bridging the Gap between Primary and Acute Care | Judah Goldstein | Canada | To emphasise that improving paramedic education as it pertains to geriatrics is a critical step | ≥ 65 years | - |
| Traumatic brain injury in older adults: do we need a different approach? | Matthew E Peters | United States | To discuss the challenges of, and potential solutions for TBI in older adults. | ≥ 75 years | - |
| Geriatric Neurologic Emergencies | Lara K. Kulchycki | United States | To highlight the special vulnerability of elderly trauma patients to neurologic injury | ≥ 65 years | - |
| Life-Threatening Injuries in Older Adults | Linda J. Scheetz, | United States | To describe the impact of life-threatening injuries in the older adult population, specifically injuries sustained in motor vehicle collisions, and how these injuries relate to anatomic and physiologic changes of ageing, the metabolic response to injury, the role of preexisting diseases and medications taken to treat these diseases, and complications. | ≥ 65 years | - |
| Clinical Characteristics and Outcome in Elderly Patients with Traumatic Brain Injury: For Establishment of Management Strategy | Hiroshi Karibe | Japan | To review clinical characteristics and the problems of TBI in the elderly, including delayed deterioration or the influences of either anticoagulant or antiplatelet to make reference to the management strategy and the outcome | ≥ 65 years | - |
| Geriatric emergency medicine: Research priorities to respond to “The Silver Boom” | Don Melady | Canada | To flag up research priorities in geriatric emergency medicine | ≥ 65 years | - |
| Prehospital Triage of Injured Older Adults: Thinking Slow Inside the Golden Hour | Timothy F. Platts-Mills | United States | To reconsider the management approach of older trauma patients by different thinking techniques | no age | - |
| Complexities of Geriatric Trauma Patients | Brett J. Monroe | United States | To educate about the different physiology of the elderly and affects of ageing | ≥ 65 years | - |
| Acute trauma and multiple injuries in the elderly population | Maurice F. Joyce | United States | To explain difficulties in the management of elderly trauma patients | ≥ 65 years | - |
| Field Triage Protocol in Elderly Trauma Patients: What Level of Care? | Jean-Pierre Tourtier | France | To emphasise the importance to transport elderly trauma patients to a level I trauma centre. | ≥ 75 years | - |
| When the Trauma Patient is Elderly | Judy Stevenson | United States | To highlight physiological changes of ageing, mechanism of injury and physiologic response to traumatic injury of elderly trauma patients. | ≥ 65 years | - |
| Trauma and the Elderly: The Real Picture of Their Health May be Blurred | Mary L. King | Australia | To discuss the hazards related to the elderly trauma patient and special considerations for healthcare professionals | ≥ 65 years | - |
| Undertriage Remains a Vexing Problem for Even the Most Highly Developed Trauma SystemsThe Need for Innovations in Field Triage | Craig D. Newgard | United States | To emphasise the need for innovations in field triage to tackle undertriage in older trauma patients | no age | - |
| Avoiding anchoring bias by moving beyond mechanical falls in geriatric emergency medicine | Guruprasad Nagaraj | Australia | To discuss the complexity of mechanical falls in geriatric trauma patients | no age | - |
| The growing challenge of major trauma in older people: a role for comprehensive geriatric assessment? | James Michael Fisher | UK | To write about the challenge of trauma in older people and the role of Comprehensive Geriatric Assessments | ≥ 75 years | - |
| A position paper: The convergence of aging and injury and the need for a Geriatric Trauma Coalition (GeriTraC) | Zara Cooper | United States | Position paper to improve geriatric trauma care through an interdisciplinary approach to (a) injury prevention, (b) transport and triage, (c) initial assessment and hospital management, and (d) transitions of care. | ≥ 65 years | - |
| Creating a Geriatric-Focused Model of Care in Trauma With Geriatric Education | Kai L. Bortz | United States | To educate about the mechanism of injury, complications of TBI and discuss geriatric models of care. | ≥65 years | - |
| Trauma in the Older Adult | Stephanie Bonne | United States | To give an overview of Epidemiology and Evolving Geriatric Trauma Principles. | ≥65 years | - |

# *The Level of Evidence was determined as suggested by the Oxford Centre for Evidence-Based Medicine: Levels of Evidence (March 2009)*


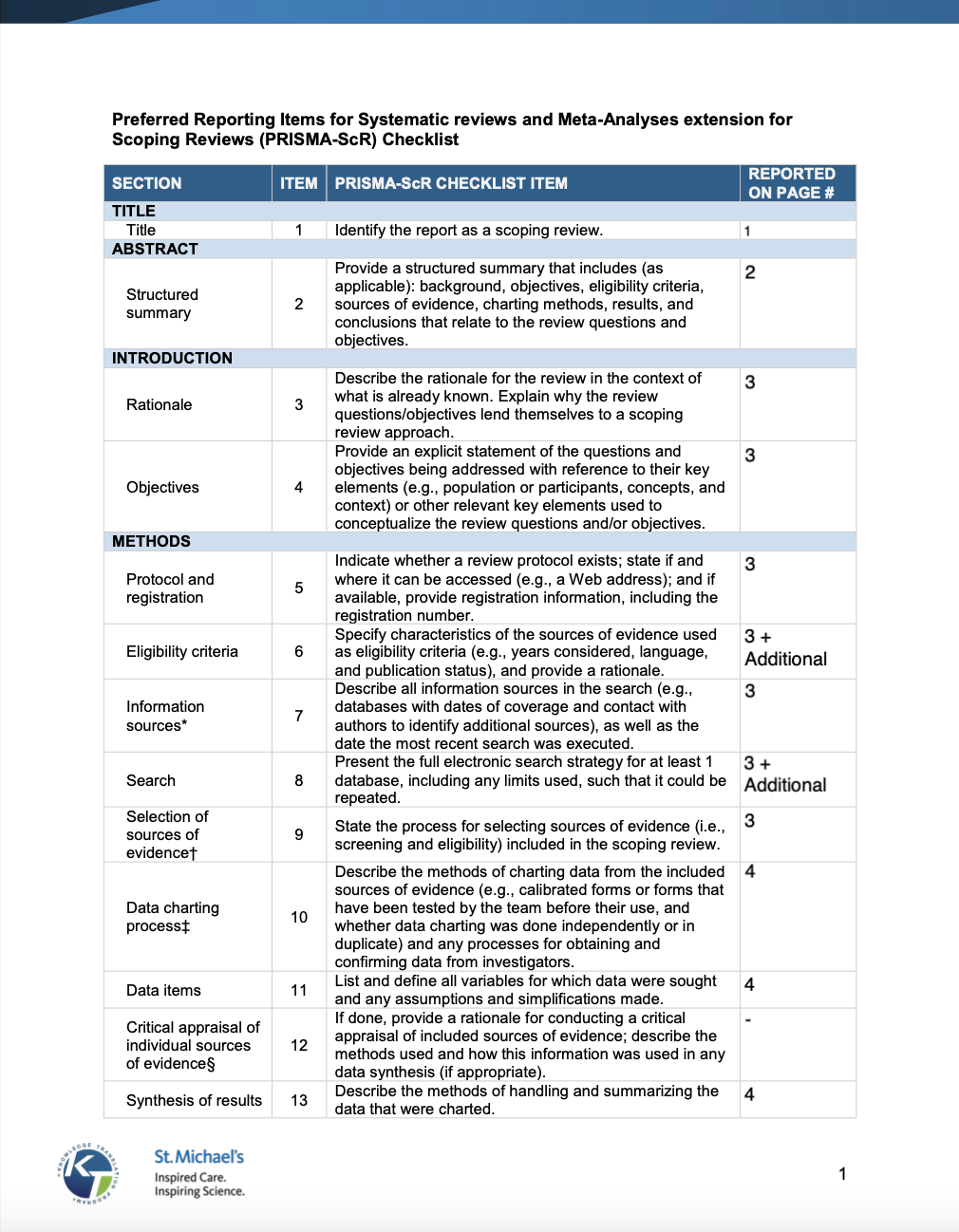


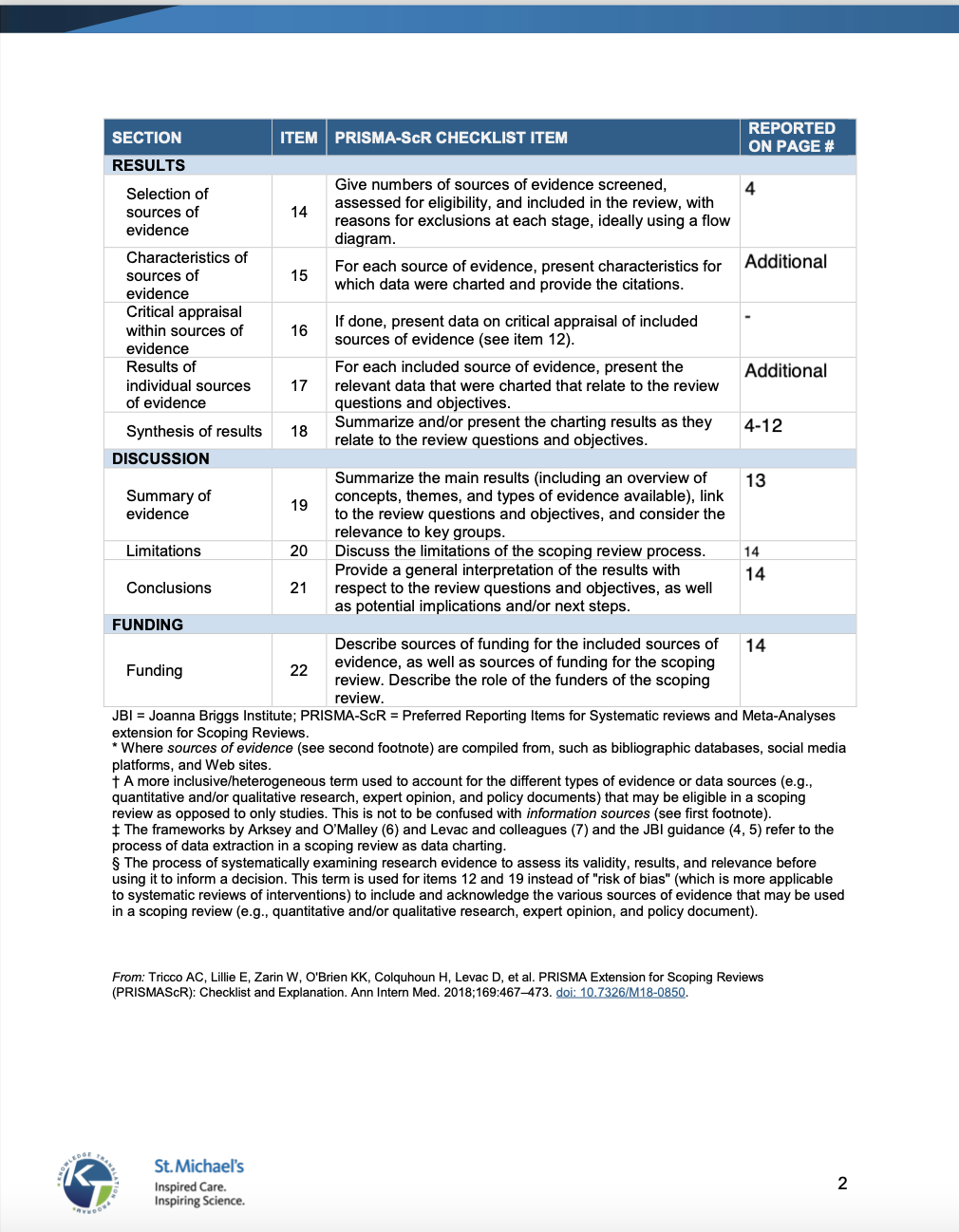

Supplement: Supplementary file 1 — Additional file 1. [file 13049_2021_922_MOESM1_ESM.docx]
